# Cortical free-water imaging in familial frontotemporal dementia associated with MAPT, GRN, and C9orf72 pathogenic variants

**DOI:** 10.21203/rs.3.rs-9677636/v1

**Published:** 2026-05-25

**Authors:** Wenqian Wu, Houyi Wu, Linyuan Zhang, Ping Dai, Wencai Ding, Xiaolei Tang, Tonghua Zhang, Zhiding Shao

**Affiliations:** 1. The Second Affiliated Hospital of Wannan Medical College, Wuhu, 241000, China

**Keywords:** frontotemporal lobar degeneration, familial frontotemporal dementia, free-water imaging, diffusion MRI, cortical microstructure, C9orf72, GRN, MAPT, neurofilament light chain

## Abstract

**Background:**

Familial frontotemporal lobar degeneration (FTLD) caused by pathogenic variants in C9orf72, GRN, and MAPT provides a unique framework for evaluating imaging markers of genotype-specific neurodegeneration. Conventional cortical mean diffusivity (cMD) is sensitive to microstructural injury but is influenced by extracellular free-water effects. We investigated whether cortical free water (cFW) provides a sensitive imaging readout of cortical microstructural alterations across the three major genetic forms of familial FTLD, and compared its spatial distribution and clinical relevance with cMD and free-water–corrected tissue mean diffusivity (MD-t).

**Methods:**

We analyzed data from 324 participants from the ARTFL-LEFFTDS Longitudinal Frontotemporal Lobar Degeneration (ALLFTD) study, including 199 carriers of pathogenic variants in C9orf72 (n = 85), GRN (n = 56), or MAPT (n = 58), spanning asymptomatic and symptomatic stages, and 125 non-carrier family members. Surface-based cortical maps of cMD, MD-t, and cFW were generated from diffusion MRI. Group differences between each genotype and non-carrier controls were assessed using general linear models adjusted for age and sex. Associations with disease severity, measured by the CDR^®^ plus NACC FTLD scale, and plasma neurofilament light chain (NfL) were examined within each genotype, with family-wise error correction for surface-based analyses.

**Results:**

Across C9orf72, GRN, and MAPT carriers, cFW showed the most spatially extensive cortical abnormalities relative to non-carrier controls, whereas MD-t effects were consistently more circumscribed than conventional cMD. This pattern was observed across all three genotypes, with particularly widespread cFW elevations in C9orf72 and GRN carriers and more frontotemporal-predominant alterations in MAPT carriers. cFW also exhibited the broadest positive associations with FTLD-CDR and plasma NfL across genotypes, while MD-t associations were more regionally restricted. These findings suggest that extracellular free-water changes contribute substantially to diffusion abnormalities in familial FTLD and provide information complementary to tissue-restricted diffusivity.

**Conclusions:**

cFW is a sensitive cross-genotype imaging marker of cortical microstructural alterations and disease burden in familial FTLD associated with C9orf72, GRN, and MAPT pathogenic variants. Compared with conventional cMD and MD-t, cFW captures more spatially extensive disease-related abnormalities and shows broader cortical associations with clinical severity and plasma NfL. Longitudinal studies are needed to determine whether cFW improves prediction of disease progression and sensitivity to change in genetic FTLD trials.

## Introduction

Frontotemporal lobar degeneration (FTLD) encompasses a spectrum of neurodegenerative disorders characterized by progressive deterioration in cognition, behavior, and language. The disease is pathologically diverse and clinically heterogeneous, involving multiple molecular and cellular pathways[1, 2]. Genetic factors play a central role in its pathogenesis, with C9orf72 repeat expansions, GRN mutations, and MAPT mutations being the three major causes of familial FTLD, together accounting for approximately 60%–80% of inherited cases[3–5]. These genetic subtypes exhibit distinct clinical phenotypes, rates of progression, and regional patterns of brain degeneration[6, 7]. However, the biological substrates that drive these genotype-specific imaging differences remain incompletely understood, including the relative contributions of proteinopathy-related neurodegeneration and neuroinflammatory processes[8–11]. Diffusion tensor imaging (DTI) offers a noninvasive approach for probing microstructural brain alterations in FTLD[12, 13]. Among DTI-derived indices, mean diffusivity (MD) quantifies the overall mobility of water molecules and serves as an indirect marker of axonal integrity and cellular density[12–14]. Although MD is sensitive to early microstructural changes in neurodegenerative disorders, it remains biologically nonspecific. Within cortical gray matter, MD demonstrates particular sensitivity to subtle alterations[15, 16]. Prior studies have linked higher cMD to neurodegeneration-related cortical vulnerability, cognitive impairment, and blood or imaging markers of disease burden, including NfL and diffusion-derived indices of glymphatic/perivascular function[17, 18].

However, conventional MD measurements are strongly affected by extracellular free water (FW). Increases in FW—arising from tissue edema, neuroinflammation, or expansion of extracellular space secondary to atrophy—may mask true intracellular diffusion abnormalities[19–22]. To overcome this limitation, FW-corrected MD (MD-t) has been proposed to better isolate intracellular microstructural injury. At the same time, FW itself represents an independent biomarker, reflecting extracellular alterations related to neuroinflammation, blood–brain barrier breakdown, or neuronal loss[23]. FW can confound conventional DTI measures and reduce the tissue specificity of MD[12, 24]. Conversely, FW itself provides pathophysiological information, serving as a sensitive indicator of inflammation, atrophy, and extracellular space expansion[13]. In Alzheimer’s disease (AD), increased FW has been associated with biomarker-defined disease stage and clinical decline.[25]

Although abnormalities in DTI metrics have been reported in FTLD, systematic comparisons of MD, MD-t, and FW across the three major genetic subtypes—C9orf72, GRN, and MAPT—remain limited. Most previous studies have examined either a single genotype or a single diffusion metric, thereby hindering a comprehensive understanding of the genotype–imaging biomarker–pathological mechanism relationship. Furthermore, the associations between these diffusion metrics and clinical disease severity, as indexed by the Frontotemporal Lobar Degeneration Clinical Dementia Rating (FTLD-CDR), or with blood biomarkers of neuronal injury such as plasma NfL, have not been systematically validated across genotypes[1, 2]. These gaps limit the translational potential of DTI-derived measures as quantitative biomarkers for disease monitoring and prognostic assessment in FTLD.

Based on these gaps, this study aimed to:(1) compare cortical MD, MD-t, and FW between carriers of the three major FTLD genotypes—C9orf72, GRN, and MAPT—and cognitively healthy controls;(2) evaluate the associations of these diffusion metrics with disease severity, as indexed by the FTLD-CDR, within each genotype; and (3) investigate their relationships with plasma NfL levels. Our overarching goal was to advance the understanding of genotype-specific microstructural alterations in FTLD, to assess the clinical utility of diffusion MRI (dMRI) metrics as quantitative biomarkers, and to clarify the spatial distribution and potential biological mechanisms underlying these changes.

## Methods

### Participants and Data Acquisition

Participants were recruited from the ALLFTD consortium, a multicenter observational cohort established to improve clinical characterization and facilitate therapeutic development in frontotemporal lobar degeneration (FTLD).[26] We included 324 individuals: 199 carriers of pathogenic variants in C9orf72 (n=85), GRN (n=56), or MAPT (n=58), and 125 non-carrier family members served as controls. Clinical diagnoses of FTLD-spectrum syndromes (when applicable) were made according to consensus criteria, and pathogenic variant status was confirmed by genetic testing.[27]

### Genetic testing

All participants underwent research-based genetic testing to determine carrier status for pathogenic variants associated with FTLD, including C9orf72, GRN, and MAPT. Detailed procedures and results are reported elsewhere (Ramos et al., this issue). While clinical genetic testing with disclosure was available to participants, most asymptomatic individuals elected not to pursue clinical testing. Research genetic results were not disclosed to participants and were masked to clinicians. Accordingly, mutation status used in the present analyses was defined exclusively based on research testing.[28, 29]

### Neuroimaging data acquisition

All MRI data were acquired on 3T scanners using an 8-channel phased-array head coil. High-resolution T1-weighted images were collected with a 3D magnetization-prepared rapid gradient echo (MPRAGE) sequence (TR=2300 ms, TE=3 ms, TI=900 ms, flip angle=8°, voxel size=1.2×1.0×1.0 mm^3^). Diffusion-weighted images were acquired in the axial plane using single-shot echo-planar imaging (EPI) (TR=10,200 ms; acquisition matrix=128×128), with 41 diffusion-weighted directions and 5 non-diffusion-weighted (b0) volumes (b=1000 s/mm^2^). Slice thickness was 2.7 mm, yielding isotropic 2.7-mm resolution.

### Cortical Mean Diffusivity and Free-Water Analysis

Cortical diffusion processing followed a previously validated surface-based pipeline.[30] Diffusion-weighted images (DWIs) were processed with a customized surface-based DTI workflow integrating FSL (v6.0.7) and FreeSurfer (v6.0). Data underwent quality control, excluding scans with head motion >2 mm translation or >2° rotation. DWIs were corrected for eddy-current–induced distortions and head motion using FSL eddy. Susceptibility-induced distortion was corrected using Synb0-DISCO with T1-weighted anatomical images as reference.[31] Diffusion tensors were fitted with FSL dtifit to derive mean diffusivity maps; cortical mean diffusivity (cMD) was obtained by sampling diffusion metrics onto the cortical surface as described below. Free-water (FW) and FW-corrected tissue mean diffusivity (MD-t) maps were estimated using a bi-tensor model implemented in MATLAB.[32–34] Using boundary-based registration (BBR),[35] FW (and MD-t) maps were aligned to each participant’s T1-weighted image, sampled at 50% cortical depth, projected onto the individual cortical surface, and mapped to the fsaverage template. Surface maps were smoothed with a 10-mm full-width-at-half-maximum Gaussian kernel.

### Clinical assessments

Disease severity was assessed using the Frontotemporal Lobar Degeneration Clinical Dementia Rating scale (CDR^®^ plus NACC FTLD)[36], a standardized instrument for staging cognitive and behavioral/functional impairment in FTLD.[37]

### Plasma neurofilament light chain (NfL)

Plasma neurofilament light chain (NfL) was measured as a biomarker of neuroaxonal injury. Blood was collected into EDTA tubes, centrifuged at 1500 × g for 15 min at 4°C, aliquoted, and stored at −80°C until analysis. Plasma NfL concentrations were quantified using the Quanterix NF-Light assay (single-molecule array [Simoa]) according to the manufacturer’s instructions. Samples were assayed in duplicate on a Quanterix HD-X Analyzer, with run-level quality control procedures implemented to ensure assay reliability.

### Statistical analysis

All statistical analyses were conducted in R (v4.4.1). Site-related variability in cortical diffusion metrics (MD, MD-t, and FW) was harmonized using neuroCombat, with MRI site modeled as the batch variable and age and sex included as covariates to be preserved.[38] Group differences in cortical MD, MD-t, and FW were evaluated using general linear models (GLMs), comparing each genetic group (MAPT, GRN, C9orf72) with the control group, while adjusting for age and sex. Associations between diffusion metrics and clinical/biomarker measures (CDR^®^ plus NACC FTLD and plasma NfL) were examined using correlation/regression analyses. Statistical significance for surface-based analyses was assessed using permutation-based Monte Carlo simulation (10,000 permutations) with family-wise error (FWE) correction; two-sided FWE-corrected P<0.01 was considered significant.

## Results

### Demographic and Clinical Characteristics

Demographic and clinical characteristics are summarized in Table 1. Groups differed significantly in age at assessment (P<0.001): GRN carriers were the oldest, followed by C9orf72 and MAPT carriers, with non-carriers representing the youngest group. Among symptomatic carriers, age at onset differed across genotypes (P<0.001); Sex distribution (P=0.883) and educational attainment (mean ≈15.5 years; P=0.993) were comparable across groups.

The distribution of primary clinical phenotypes differed markedly by genotype (P<0.001). Approximately half of C9orf72 and GRN carriers were clinically asymptomatic; among symptomatic participants, behavioral-variant FTD (bvFTD) was the most common presentation. MAPT carriers showed the highest proportion of bvFTD. All non-carriers were clinically normal. Cognitive and functional performance also differed across groups: MoCA differed by group (median [range]; P = 0.003), with lower scores in mutation carriers (C9orf72: 26.0 [0–30]; GRN: 27.0 [2–30]; MAPT: 27.0 [6–30]) than in non-carriers (28.0 [19–30]), and MAPT carriers demonstrated greater impairment across multiple domains, including memory, orientation, judgment, and communication (all P<0.001). Disease severity as indexed by the CDR^®^ plus NACC FTLD scale differed across carrier groups, with GRN carriers showing greater impairment than C9orf72 and MAPT carriers. Plasma neurofilament light chain (NfL) concentrations also differed (P<0.001), highest in GRN and C9orf72, intermediate in MAPT, and lowest in non-carriers.

### Cortical alterations in MD, MD-t, and FW across FTLD genotypes

We compared cortical MD, tissue-corrected MD (MD-t), and free-water (FW) between each pathogenic-variant group and non-carrier controls (Figure 1). In C9orf72 carriers, MD increases were widespread, involving much of the frontal and occipital cortices and extending to the parieto-occipital junction, inferior parietal lobule, cuneus, and lingual gyrus. In contrast, MD-t abnormalities were more spatially restricted, primarily affecting the bilateral occipital cortex and left frontal regions. FW showed the most extensive involvement, with elevations across nearly the entire cortex, sparing the precentral and postcentral gyri.

In GRN carriers, MD increases were prominent across large portions of the frontal, temporal, and occipital lobes, with additional involvement of the insula and cingulate cortex. MD-t abnormalities were again more circumscribed, confined to focal frontal and temporal regions. FW elevations were widespread, spanning almost the entire cortical mantle and extending into subcortical areas.

In MAPT carriers, MD increases were comparatively limited, primarily involving the superior and middle frontal gyri and inferior-to-middle temporal cortices, with even more restricted MD-t alterations. In contrast, FW elevations extended bilaterally across frontal and temporal cortices, including the insula, transverse temporal cortex, and inferior parietal regions, suggesting that extracellular free-water increases contribute substantially to the observed diffusivity changes in this group.

### Associations with disease severity

We next examined associations between cortical diffusion metrics (MD, MD-t, and FW) and disease severity indexed by the FTLD-CDR (CDR^®^ plus NACC FTLD) across genotypes (Figure 2). In C9orf72 carriers, MD showed significant positive associations with FTLD-CDR, most prominently in frontal regions (including the orbital portion of the inferior frontal gyrus) and extending to occipital and parietal cortices. These associations were substantially attenuated for MD-t, with only limited effects in the frontal poles, posterior occipital cortex, and superior temporal gyrus. In contrast, FW showed a markedly broader association pattern, spanning most of the frontal, temporal, occipital, and parietal cortices.

In GRN carriers, MD–FTLD-CDR associations were widespread, involving nearly the entire cortex with relative sparing of small regions in the precentral and lingual gyri. FW exhibited an even more extensive pattern. By comparison, MD-t associations were more circumscribed, primarily involving superior frontal and superior parietal regions, the precuneus, supramarginal gyrus, lateral occipital cortex, and middle temporal cortex.

In MAPT carriers, both MD and FW were significantly associated with FTLD-CDR, with FW showing a broader distribution that extended beyond MD-associated regions to include the precentral and postcentral gyri, middle frontal cortex, and superior parietal cortex. MD-t also showed significant associations—mainly within the middle frontal, inferior parietal, and cingulate regions—but with substantially reduced spatial extent relative to MD and FW.

### Associations with NfL

We finally examined associations between cortical diffusion metrics and NfL levels across genotypes (Figure 3). In C9orf72 carriers, MD showed significant positive associations with NfL, primarily involving the bilateral frontal poles, posterior middle frontal gyrus, frontal opercular/triangular regions, precuneus, and posterior cingulate cortex, with additional involvement of lateral occipital and transverse temporal cortices. FW demonstrated a substantially more extensive association pattern, extending into the inferior and superior parietal lobules and the supramarginal gyrus, whereas MD-t associations were more circumscribed and largely confined to the bilateral middle frontal, cingulate, and inferior temporal regions.

In GRN carriers, MD–NfL associations were limited, mainly involving the bilateral frontal poles and inferior frontal gyrus. In contrast, FW showed widespread associations across much of the frontal and temporal cortices; a similar but attenuated pattern was observed for MD-t.

In MAPT carriers, MD exhibited widespread positive associations with NfL across frontal, temporal, parietal, and occipital cortices. FW showed an even broader distribution, additionally involving the precentral and postcentral gyri. MD-t associations remained significant but were spatially restricted, predominantly within frontal regions.

## Discussion

This study systematically characterized cortical MD, MD-t, and FW alterations across the three major FTLD genotypes (C9orf72, GRN, and MAPT), and examined their associations with disease severity and plasma NfL levels. Across all genotypes, cortical free water (cFW) showed the most spatially extensive abnormalities, exceeding the distributions observed for conventional mean diffusivity (MD) and free-water–corrected tissue diffusivity (MD-t). Moreover, cFW exhibited the broadest associations with both clinical disease severity (FTLD-CDR) and plasma NfL, supporting its potential as a sensitive imaging readout of disease burden that generalizes across major genetic forms of FTLD.

A parsimonious interpretation of these convergent patterns is that extracellular water compartment changes contribute substantially to diffusion abnormalities in genetic FTLD. Conventional MD reflects a mixture of tissue and extracellular components, whereas MD-t is designed to isolate tissue-restricted diffusivity by accounting for the free-water fraction. The consistent finding that MD-t effects were more circumscribed than MD—together with the widespread cFW elevations—suggests that a large portion of apparent MD increases may be driven by increases in extracellular free water rather than tissue-only microstructural disruption. Biologically, elevated cFW is compatible with several non-mutually exclusive processes relevant to FTLD, including expansion of interstitial space associated with neurodegeneration and atrophy, neuroinflammatory or glial responses, and changes in perivascular or extracellular fluid dynamics. Importantly, while cFW appears highly sensitive, it is not pathology-specific, and its mechanistic underpinnings in FTLD will require multimodal validation.

### The regional distributions of MD, MD-t, and FW differed among the three genotypes, reflecting their distinct molecular and cellular pathological substrates.

In the C9orf72 genotype, MD abnormalities extended widely across cortical regions, whereas FW alterations were even more extensive, with MD-t abnormalities reduced in spatial extent. This pattern is consistent with the multisystem involvement characteristic of C9orf72 repeat expansions, which may lead to diffuse structural brain damage through mechanisms such as RNA toxicity, dipeptide repeat protein deposition, and neuronal loss.[3, 8, 39–41] Neuroimaging studies of C9orf72 carriers have similarly reported widespread cortical involvement across multiple lobes.[7] Taken together, these observations suggest that FW contributions may substantially affect the spatial extent of MD abnormalities in C9orf72-related FTLD, and that MD-t may provide complementary information that is relatively more tissue-restricted than conventional MD.

In the GRN genotype, MD abnormalities were prominent across frontal, temporal, and occipital regions, whereas FW increases were more widespread, with MD-t abnormalities markedly attenuated. GRN mutations result in progranulin deficiency, causing lysosomal dysfunction and neuroinflammation.[9] The widespread FW elevation may be compatible with inflammation-related tissue changes and extracellular space expansion.[10] In contrast, the more localized distribution of MD-t abnormalities suggests that tissue-restricted microstructural disruption may be comparatively circumscribed in this genotype, further supporting FW as a sensitive marker of genotype-related disease burden.

In the MAPT genotype, MD abnormalities were the most spatially restricted, primarily involving frontal and temporal regions, while MD-t changes were even more limited. In contrast, FW exhibited pronounced increases extending across bilateral frontal and temporal cortices, with additional parietal involvement. MAPT mutations lead to abnormal tau protein aggregation, primarily affecting neurons and axons within frontotemporal networks—consistent with the localized pattern of MD abnormalities.[4] The extensive FW elevations, however, may reflect downstream processes such as neuronal loss, gliosis, and extracellular space expansion induced by tau pathology.[11] These results indicate that even in predominantly tau-driven pathology, FW can detect more widespread changes than MD or MD-t, highlighting its sensitivity to disease-related microstructural alterations.

In summary, across the three FTLD genotypes, MD, MD-t, and FW each displayed distinct spatial patterns, corresponding to their unique pathological substrates. Among these diffusion metrics, FW consistently demonstrated the most extensive alterations, emphasizing its potential utility as a cross-genotype biomarker of cortical microstructural change in FTLD.

### Association with Disease Severity (FTLD-CDR): genotype-specific patterns and the extent of FW effects

This study demonstrated that FW exhibited broader and more spatially extensive associations with FTLD-CDR than either MD or MD-t, with clear genotype-specific patterns. In C9orf72 and GRN carriers, FW–CDR associations covered substantially larger cortical territories than MD—most prominently across frontotemporal and occipital regions in C9orf72 and across widespread cortical regions in GRN—consistent with FW capturing global disease-related processes such as neurodegeneration and atrophy that align with the FTLD-CDR’s emphasis on overall functional decline rather than isolated axonal microstructural injury. In contrast, MD-t associations were weaker but detectable, supporting the notion that FW indexes more global (“macro-level”) disease burden, whereas MD-t reflects more localized, tissue-restricted change. In MAPT carriers, FW associations overlapped with MD-associated regions but additionally extended into sensorimotor cortices (precentral and postcentral gyri), suggesting that FW may capture both cognitive deterioration and broader network involvement, whereas MD appears comparatively restricted.[6] Consistent with our prior work showing stronger MD–FTLD-CDR coupling than cortical thickness,[1] the present results further indicate that FW may provide an even more sensitive quantitative marker for disease staging and monitoring in FTLD.

### Association with plasma NfL: concordance between FW and neuroaxonal injury

Neurofilament light chain (NfL) is a key cytoskeletal protein, and elevated plasma NfL is widely used as a biomarker of neuroaxonal injury and disease burden across neurodegenerative disorders.[42] In the present study, FW–NfL associations were more extensive and robust than those observed for MD or MD-t across all three genotypes, supporting a close link between extracellular free-water changes and neuroaxonal injury burden. In C9orf72 carriers, FW–NfL associations extended into parietal regions beyond the more restricted MD pattern, consistent with diffuse multiregional involvement. In GRN carriers, MD–NfL associations were highly limited, whereas FW showed broader frontotemporal associations, compatible with prominent extracellular-compartment changes accompanying GRN-related disease processes. In MAPT carriers, FW not only overlapped with MD-associated regions but also extended into sensorimotor cortices (precentral and postcentral gyri), consistent with broader network involvement. Across genotypes, MD-t maintained significant but spatially restricted associations with NfL, underscoring the complementary nature of these metrics: FW appears most sensitive to widespread disease-related extracellular alterations, whereas MD-t may reflect more tissue-restricted microstructural disruption that is less spatially extensive.

### Clinical Implications and Limitations

The present findings have important implications for the precision diagnosis and management of FTLD. Among the diffusion-derived metrics, FW emerged as a cross-genotype biomarker for monitoring disease severity, demonstrating robust associations with both clinical ratings (FTLD-CDR) and the plasma biomarker NfL. Its consistent sensitivity across genotypes suggests potential value for tracking disease progression and treatment response in clinical settings. MD-t, in contrast, showed particular strength in detecting axonal injury—especially in *MAPT*-related FTLD—whereas MD provided a more general indicator of global microstructural abnormalities. Together, these metrics form a complementary framework that may guide genotype-specific disease profiling, improve patient stratification in clinical trials, and inform the development of individualized therapeutic strategies for this heterogeneous disorder.

The distinct spatial patterns of MD, MD-t, and FW across the *C9orf72, GRN*, and *MAPT* genotypes underscore the necessity of integrating genotype-specific neuroimaging signatures into clinical assessment and prognostic models. Such integration may enhance the prediction of cognitive decline, disease progression, and treatment efficacy, ultimately advancing precision medicine approaches in FTLD.

Several limitations should be acknowledged. First, the sample size was modest; although we compared three genetic subtypes, the number of patients per group remained limited, warranting replication in larger, multicenter cohorts. Second, the cross-sectional design precluded the assessment of longitudinal dynamics between diffusion metrics, clinical progression, and functional decline. Future longitudinal and interventional studies will be essential to establish causal relationships and determine the prognostic value of these biomarkers. Third, all diffusion parameters were derived from a single b-value acquisition, which may limit the precision of FW and MD-t estimation. Incorporating multi–b-value or multi-shell diffusion imaging in future work will further enhance the accuracy, reproducibility, and biophysical interpretability of these metrics.

Future studies should test whether within-person longitudinal changes in cFW predict subsequent clinical progression and changes in plasma NfL, and whether cFW improves sensitivity to change relative to conventional diffusion metrics. Integrating cFW with complementary modalities—such as cortical thickness, perfusion/vascular imaging, or molecular PET where available—may help clarify biological drivers and enhance interpretability. If validated longitudinally, cFW could represent a practical imaging endpoint for genetic FTLD trials aimed at detecting subtle disease-related changes across heterogeneous genotypes and stages.

## Figures and Tables

**Figure 1.** Genotype-specific group differences in cortical microstructural measures. Surface-based statistical maps depict significant vertex-wise group differences in cortical mean diffusivity (cMD; top row), free-water–corrected mean diffusivity (MD-t; middle row), and cortical free water (cFW; bottom row) comparing each pathogenic-variant group (C9orf72, GRN, MAPT) with non-carrier controls. Columns correspond to contrasts (left: C9orf72 vs controls; middle: GRN vs controls; right: MAPT vs controls). C9orf72 carriers showed widespread increases in cMD and cFW across bilateral frontal and parietal cortices. GRN carriers demonstrated predominantly posterior alterations, whereas MAPT carriers exhibited more focal abnormalities in orbitofrontal and temporal regions. Clusters survived correction for multiple comparisons using cluster-wise family-wise error control based on Monte Carlo simulation (10,000 iterations; FreeSurfer), with a significance threshold of P<0.01.

**Figure 2.** Associations between cortical microstructural measures and disease severity across FTLD genotypes. Surface-based statistical maps depict significant vertex-wise associations between FTLD-CDR (CDR^®^ plus NACC FTLD) scores and cortical mean diffusivity (cMD; top row), free-water–corrected mean diffusivity (MD-t; middle row), and cortical free water (cFW; bottom row) within each pathogenic-variant group. Columns correspond to genotype (left: C9orf72; middle: GRN; right: MAPT). In C9orf72 carriers, cFW showed the most spatially extensive associations, involving frontal and temporal cortices with additional parietal involvement. GRN carriers exhibited widespread associations across frontal, temporal, and parietal regions. In MAPT carriers, cFW associations overlapped with cMD-associated regions and additionally extended into sensorimotor cortices. Clusters survived correction for multiple comparisons using cluster-wise family-wise error control based on Monte Carlo simulation (10,000 iterations; FreeSurfer), with a significance threshold of P<0.01.

**Figure 3.** Associations between cortical microstructural measures and plasma NfL across FTLD genotypes. Surface-based statistical maps depict significant vertex-wise associations between plasma NfL and cortical mean diffusivity (cMD; top row), free-water–corrected mean diffusivity (MD-t; middle row), and cortical free water (cFW; bottom row) within each pathogenic-variant group. Columns correspond to genotype (left: C9orf72; middle: GRN; right: MAPT). Across genotypes, cFW showed the most spatially extensive associations with NfL, whereas cMD and MD-t exhibited more restricted patterns. Clusters survived correction for multiple comparisons using cluster-wise family-wise error control based on Monte Carlo simulation (10,000 iterations; FreeSurfer), with a significance threshold of P<0.01.
